# 

**Published:** 1896-08-29

**Authors:** 


					THE HOSPITAL. j , ABSOLUTELY PURE, therefore BEST,
August 29th, 1890.
CADBURY'S
COCOA
NO ALKALIES USED.
*
asco? Western Inhomart " ?^^nomolhakdMORWICH ho&bisal
No. sisVYoi. xx. WITH EXTRA NURSING SUPPLEMENT. ?gEL.SEKHff-
Satttdtuv A irnTTQT 9Qttt 1 RQfi [Registered at the General Post Offioe as a Newspaper for Mont lily Parte, 6d.; post free, 8i
) AUGUST aJi II, loyo. transmission in the United Kingdom and Abroad,] Ditto per Annum, 8s. 6d., post free.

				

